# PIC-RNA-seq for Region-Specific Transcriptomic Analysis of Chicken Limb Bud Progenitors

**DOI:** 10.21769/BioProtoc.5843

**Published:** 2026-10-20

**Authors:** Seung June Kwon, Zhaonan Zou, Seiko Watanabe, Mizuki Honda, Shinya Oki, Yuji Atsuta

**Affiliations:** 1 Graduate School of Systems Life Sciences, Kyushu University, Fukuoka, Japan; 2 Institute of Resource Development and Analysis, Kumamoto University, Kumamoto, Japan; 3 Graduate School of Integrated Sciences for Life, Hiroshima University, Higashi-Hiroshima, Japan

**Keywords:** PIC-RNA-seq, Chicken embryo, Limb development, Limb bud progenitor cell, Somatic lateral plate mesoderm

## Abstract

Region-specific RNA sequencing is a powerful approach for investigating tissue differentiation and dynamic changes in gene expression during embryonic development. The chicken embryo has long served as an important model system in developmental biology. However, the limited availability of tissue-specific reporter lines makes region-specific RNA-seq approaches particularly valuable in this organism. Here, we applied photo-isolation chemistry-based RNA sequencing (PIC-RNA-seq) to the somatic lateral plate mesoderm (sLPM) before the emergence of limb bud progenitor cells (LPCs) and to early LPCs in chicken embryos. These analyses revealed the upregulation of multiple genes, including *Hox* genes, in LPCs, suggesting the initiation of their regional patterning program. This workflow enables visualization of dynamic changes in the gene expression profile of LPCs and should also be applicable to other tissues in avian embryos.

Key features

• An optimized PIC-RNA-seq protocol for the somatic lateral plate mesoderm (sLPM) and limb bud progenitor cells (LPCs) in chicken embryos.

• Applicable to chicken embryos without the need for tissue-specific transgenic reporter lines.

• Robust generation of high-quality RNA-seq datasets with sufficient sequencing depth, unique molecular identifiers (UMIs), and detected genes for downstream analyses.

• Enables direct comparison between pre-specified sLPM and specified LPCs, allowing the detection of transcriptional changes accompanying early LPC specification.

## Graphical overview


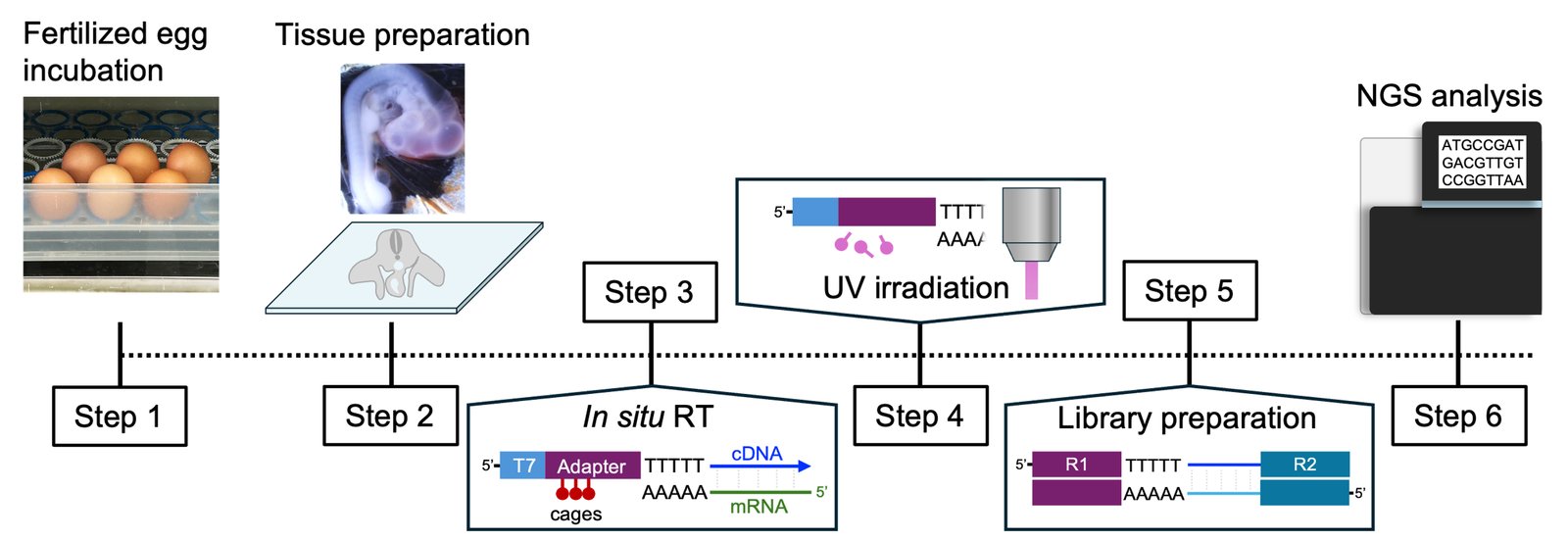


## Background

Embryonic development is orchestrated by spatiotemporally regulated gene expression programs that drive cell fate specification, tissue patterning, and organogenesis. Identifying transcriptomic changes within anatomically defined regions is therefore essential for understanding the molecular basis of developmental processes. In particular, the transition from multipotent progenitor populations to lineage-committed cells often occurs within a narrow developmental window, requiring methods that provide both high spatial resolution and robust transcriptomic profiling.

The chicken embryo has long served as a classical model for developmental biology because of its accessibility for in ovo manipulation and live imaging. Despite these advantages, transcriptomic analysis of spatially restricted embryonic tissues remains technically challenging. Fluorescence-based cell isolation generally requires tissue-specific transgenic reporter lines, which are still limited in chickens. Manual microdissection can enrich target tissues but often includes surrounding cells, whereas single-cell RNA sequencing requires tissue dissociation, resulting in the loss of spatial information and introducing additional technical complexity. Integration of spatial transcriptomics with single-cell RNA-seq can provide spatial information on transcriptionally defined cell populations but typically requires additional single-cell datasets and computational mapping or deconvolution, rather than direct transcriptomic profiling of a microscopically defined region of interest (ROI). Laser capture microdissection (LCM) coupled with RNA-seq also enables transcriptomic analysis of microscopically defined regions in tissue sections [1]. However, the spatial resolution and shape of the ROI are constrained by laser-cutting precision and the requirement for physical dissection and recovery of the target tissue. Although LCM facilitates the separate collection and analysis of multiple ROIs from the same tissue section, physical recovery of very small or complexly shaped regions can be technically challenging.

PIC-RNA-seq overcomes many of these limitations by defining ROIs through patterned light irradiation rather than physical tissue isolation [2,3]. The method uses photo-caged primers for in situ reverse transcription, followed by patterned light irradiation to uncage cDNAs specifically within selected ROIs. Only cDNAs from the irradiated regions become competent for subsequent amplification, enabling spatially selective transcriptomic profiling. This approach allows flexible selection of small or irregularly shaped regions at optical resolution. Because tissue selection is performed directly on tissue sections, the method can be applied without tissue dissociation or tissue-specific transgenic reporter lines, making it particularly well suited for developmental studies in organisms with limited genetic resources. We previously employed this approach to investigate the molecular basis of avian sternal diversification [4].

In this protocol, we adapted the original PIC-RNA-seq workflow with minor modifications for the analysis of the somatic lateral plate mesoderm (sLPM) before limb bud progenitor cell (LPC) specification and of specified LPCs in chicken embryos. The optimized workflow consistently yields high-quality sequencing data from small embryonic regions and enables direct comparison of closely related developmental stages. Although demonstrated here using the early limb field, this protocol should be broadly applicable to transcriptomic studies of other spatially defined tissues in avian embryos.

## Materials and reagents

**Biological materials**

1. Fertilized white leghorn chicken *Gallus gallus* eggs (purchased from Yamagishi poultry farm)

2. Fresh-frozen cryosections (10 μm) of chicken (*Gallus gallus*) embryos at Hamburger–Hamilton (HH) stages 14 and 19, mounted on MAS-coated slides

**Reagents**

1. Isopentane (Nacalai Tesque, catalog number: 26404-75)

2. OCT compound (Sakura Finetek, catalog number: 4583)

3. Nuclease-free water (Nacalai Tesque, catalog number: 06442-95)

4. Distilled water (Nacalai Tesque, catalog number: 49506-64)

5. Phosphate-buffered saline (PBS) tablets, pH 7.4 (Takara Bio, catalog number: T9181)

6. 16% formaldehyde (w/v), methanol-free (Electron Microscopy Sciences, catalog number: 15710)

7. Tris-EDTA (TE) buffer solution, pH 8.0 (Nacalai Tesque, catalog number: 32739-31)

8. 0.1 M DTT (Thermo Fisher Scientific, catalog number: Y00147)

9. Proteinase K solution, 20 mg/mL (Kanto Chemical, catalog number: 34060-96)

10. 5 M NaCl (Nacalai Tesque, catalog number: 06900-14)

11. Polyethylene glycol 8,000, 50% (w/v) (Nacalai Tesque, catalog number: 26065-54)

12. 0.5 M EDTA, pH 8.0 (Nacalai Tesque, catalog number: 06894-85)

13. Ethanol (Nacalai Tesque, catalog number: 14713-95)

14. NPOM-caged-dT-CE phosphoramidite (Glen Research, catalog number: 10-1534-95)

15. SuperScript II reverse transcriptase, 200 U/μL, with 5× first-strand buffer and 0.1 M DTT (Thermo Fisher Scientific, catalog number: 18064071)

16. RNaseOUT, 40 U/μL (Thermo Fisher Scientific, catalog number: 10777019)

17. dNTP mix, 10 mM each (New England Biolabs, catalog number: N0447L)

18. MinElute PCR Purification kit (QIAGEN, catalog number: 28006)

19. 5× second-strand buffer (Thermo Fisher Scientific, catalog number: 10812014)

20. *E. coli* DNA polymerase I, 10 U/μL (Thermo Fisher Scientific, catalog number: 18010025)

21. *E. coli* DNA ligase, 10 U/μL (Thermo Fisher Scientific, catalog number: 18052019)

22. RNase H, 2 U/μL (Thermo Fisher Scientific, catalog number: 18021071)

23. MEGAscript T7 Transcription kit (Thermo Fisher Scientific, catalog number: AMB13345), which includes ATP, GTP, CTP, UTP, 10× T7 reaction buffer, 10× enzyme mix, and TURBO DNase

24. AMPure XP beads (Beckman Coulter, catalog number: A63881)

25. RNAClean XP beads (Beckman Coulter, catalog number: A63987)

26. Phusion High-Fidelity PCR master mix (New England Biolabs, catalog number: M0531L)

27. Ribonuclease A solution, 10 mg/mL (Nacalai Tesque, catalog number: 30100-31)

28. Nuclear Violet LCS1 (AAT Bioquest, catalog number: 17543)

29. SlowFade Diamond (Thermo Fisher Scientific, catalog number: S36963)

30. PhiX Control v3 (Illumina, catalog number: FC-110-3001)

31. High-sensitivity D5000 ScreenTape (Agilent Technologies, catalog number: 5067-5592)

32. High-sensitivity D5000 reagents (sample buffer and ladder) (Agilent Technologies, catalog number: 5067-5593)

33. Tween-20 (Nacalai Tesque, catalog number: 28353-85), used as a 10% (v/v) stock in the ProK lysis solution

34. Silicone base (Shin-Etsu Chemical, catalog number: KE-106)

35. Curing agent (Shin-Etsu Chemical, catalog number: CAT-RG)

36. Activated charcoal powder (FUJIFILM, catalog number: 037-02115)

**Oligonucleotides**

The primer design follows Honda et al. [2]. All sequences are represented in the 5′–3′ direction.

1. NPOM-caged RT primer:

GCCGGTAATACGACTCACTATAGGGtttGAGttCtACAGTCCGACGATCNNNNNN[BC]ttTTTTTTTTTTTTTTTTTTTTTTV, where T = unmodified deoxythymidine, t = NPOM-caged dT, N = UMI, [BC] = the 6-nt sample barcode, and V = A, C, or G (a 3′ anchor base). The barcodes used here were CATGAG, AGGATC, AGTGCA, and TCGAAG (one per biological replicate). The caged oligonucleotide was custom-synthesized from NPOM-caged dT-CE phosphoramidite (Reagent list, #14) and OPC-purified by Nihon Gene Research Laboratory. Store at -20 °C, protected from light

2. Read2+N6 primer: GCCTTGGCACCCGAGAATTCCANNNNNN (N = random hexamer)

3. Read1 index primer: AATGATACGGCGACCACCGAGATCTACAC[i5]GTTCAGAGTTCTACAGTCCGA (replace [i5] with the appropriate Illumina index)

4. Read2 index primer: CAAGCAGAAGACGGCATACGAGAT[i7]GTGACTGGAGTTCCTTGGCACCCGAGAATTCCA (replace [i7] with the appropriate Illumina index)

*Note: Each pooled library was tagged with a unique dual index (UDI): i5 = CTGCACAA and i7 = TCTCACCT for the HH14 library, and i5 = GTGGTCTT and i7 = GTTCGAGA for the HH19 forelimb bud library. The i5 and i7 sequences listed are those entered into the sequencing sample sheet.*

**Solutions**

1. PBS (see Recipes)

2. 4% PFA in PBS (see Recipes)

3. NPOM-caged RT primer mix (see Recipes)

4. First-strand mix (see Recipes)

5. Second-strand mix (see Recipes)

6. Beads binding buffer (see Recipes)

7. IVT mix (see Recipes)

8. aRNA–N6 primer mix (see Recipes)

9. Reverse transcription mix (see Recipes)

10. Read1 and Read2 index primer mix (see Recipes)

11. Library PCR mix (see Recipes)

12. Proteinase K lysis solution (ProK solution) (see Recipes)

**Recipes**

*Note: Prepare all solutions fresh before use unless otherwise specified. For the reaction mixes, “Final concentration” refers to the concentration in the complete reaction after the component mixes are combined (e.g., the first-strand mix combined with the NPOM-caged RT primer mix, or the IVT mix added to the bead suspension).*

**1. 10× PBS**

**Table:** 

Reagent	Final concentration	Quantity or volume
NaCl	80 g/L	80 g
KCl	2 g/L	2 g
Na_2_HPO_4_	11.5 g/L	11.5 g
KH_2_PO_4_	2 g/L	2 g
Distilled water	n/a	to 1 L
Total	10×	1 L

**2. 4% PFA in PBS**

**Table:** 

Reagent	Final concentration	Quantity or volume
16% formaldehyde	4%	2.5 mL
PBS	1×	7.5 mL
Total	n/a	10 mL

Work in a fume hood.

**3. NPOM-caged RT primer mix (per section)**

**Table:** 

Reagent	Final concentration	Quantity or volume
Nuclease-free water	n/a	5 μL
NPOM-caged RT primer (500 ng/μL)	25 ng/μL	0.5 μL
dNTP mix (10 mM each)	0.5 mM	0.5 μL
Total	n/a	6 μL

Keep shielded from light.

**4. First-strand mix (per section)**

**Table:** 

Reagent	Final concentration	Quantity or volume
5× first-strand buffer	1×	2 μL
0.1 M DTT	0.01 M	1 μL
RNaseOUT (40 U/μL)	2 U/μL	0.5 μL
SuperScript II RT (200 U/μL)	10 U/μL	0.5 μL
Total	n/a	4 μL

**5. Second-strand mix (per tube)**

**Table:** 

Reagent	Final concentration	Quantity or volume
5× first-strand buffer	0.5×	2 μL
5× second-strand buffer	0.5775×	2.31 μL
dNTP mix (10 mM each)	0.115 mM	0.23 μL
*E. coli* DNA polymerase I (10 U/μL)	0.15 U/μL	0.3 μL
*E. coli* DNA ligase (10 U/μL)	0.04 U/μL	0.08 μL
RNase H (2 U/μL)	0.008 U/μL	0.08 μL
Total	n/a	5 μL

**6. Beads binding buffer**

**Table:** 

Reagent	Final concentration	Quantity or volume
50% (w/v) PEG 8,000	20%	4 mL
5 M NaCl	2.5 M	5 mL
Nuclease-free water	n/a	1 mL
Total	n/a	10 mL

Stable at room temperature (23–25 °C) for at least 1 year.

**7. IVT mix (per tube)**

**Table:** 

Reagent	Final concentration	Quantity or volume
ATP	7.5 mM	1.6 μL
GTP	7.5 mM	1.6 μL
CTP	7.5 mM	1.6 μL
UTP	7.5 mM	1.6 μL
10× T7 reaction buffer	1×	1.6 μL
10× enzyme mix	1×	1.6 μL
Total	n/a	9.6 μL

**8. aRNA–N6 primer mix (per tube)**

**Table:** 

Reagent	Final concentration	Quantity or volume
aRNA (from IVT/purification)	n/a	4.5 μL
Read2+N6 primer (250 ng/μL)	25 ng/μL	1 μL
dNTP mix (10 mM each)	0.5 mM each	0.5 μL
Total	n/a	6.0 μL

**9. Reverse transcription mix (per tube)**

**Table:** 

Reagent	Final concentration	Quantity or volume
5× first-strand buffer	1×	2 μL
0.1 M DTT	0.01 M	1 μL
RNaseOUT (40 U/μL)	2 U/μL	0.5 μL
SuperScript II RT (200 U/μL)	10 U/μL	0.5 μL
Total	n/a	4 μL

**10. Read1 and Read2 index primer mix (10 μM each)**

**Table:** 

Reagent	Final concentration	Quantity or volume
100 μM Read1 index primer	10 μM	10 μL
100 μM Read2 index primer	10 μM	10 μL
Nuclease-free water	n/a	80 μL
Total	n/a	100 μL

Stable at -20 °C for at least 1 year.

**11. Library PCR mix (per tube)**

**Table:** 

Reagent	Final concentration	Quantity or volume
Nuclease-free water	n/a	10.4 μL
Read1 and Read2 index primer mix (10 μM each)	0.8 μM each	3.6 μL
Phusion High-Fidelity PCR master mix (2×)	1×	22.5 μL
Secondary RT products	n/a	8.5 μL
Total	n/a	45 μL

**12. ProK solution**

**Table:** 

Reagent	Final concentration	Quantity or volume
1× PBS	n/a	970 μL
Tween-20 (10% (v/v) stock)	0.1%	10 μL
Proteinase K (20 mg/mL)	0.4 mg/mL	20 μL
Total	n/a	1 mL

**Laboratory supplies**

1. MAS-coated glass slides (Matsunami, catalog number: MAS-01)

2. Round coverslips, 15 mm (Matsunami, catalog number: C015001)

3. PAP pen (BMS, catalog number: BC-PAPPEN-S)

4. Humidified chamber (Cosmo Bio, catalog number: 10HTLS)

5. Handmade silicone-bottom dish: Mix 500 g of silicone base with 50 g of curing agent. Gradually add activated charcoal powder while mixing until opaque (approximately 3–4 tablespoons for a 5-mm-thick layer). Pour into a glass Petri dish, remove air bubbles by briefly applying hot air every 10 min, and leave on a level surface until completely cured (approximately 24 h at room temperature)

6. Handmade aluminum foil cryomold

7. Fine forceps (#55) (Dumont, catalog number: 11255-20)

8. 10 mL syringe (Terumo, catalog number: SS-10SZ)

9. 18G × 1.5” syringe needle (Terumo, catalog number: NN-1838R)

10. 100 mL beaker (Sanpla, catalog number: 01021)

11. Dry ice

12. Curved scissors (AZ ONE, catalog number: 2-540-11)

13. Perforated spoon (Urin Seisakujyo)

## Equipment

1. Fluorescence microscope (Leica Microsystems, model: DM6B)

2. Digital mirror device (DMD) (Mightex Systems, model: Polygon1000-G)

3. Dichroic mirror for DMD (Semrock, catalog number: Di02-R442-25336)

4. LED light source for DMD, ~365 nm, 3 W (Prizmatix, model: UHP-F-365LED)

5. Hybridization oven (TAITEC, model: HB-80)

6. Heat shaker (IKA, model: MATRIX Orbital)

7. Aspirator (AXEL, catalog number: 1-6198-01)

8. Magnetic stand (FastGene, catalog number: FG-SSMAG3.2)

9. TapeStation (Agilent Technologies, model: 4200)

10. Illumina NovaSeq 6000 sequencer (Illumina)

11. Cryostat (Thermo Scientific, model: Cryostar NX70)

12. Thermal cycler (Applied Biosystems, model: MiniAmp)

13. Vortex mixer (Scientific Industries, model: Vortex-Genie 2)

14. Benchtop centrifuge (Genereach, model: cubee-O)

## Software and datasets

1. UMI-tools (version 1.0.0) [5]; https://github.com/CGATOxford/UMI-tools (free)

2. Trim Galore! (The Babraham Bioinformatics group, version 0.6.6); https://www.bioinformatics.babraham.ac.uk/projects/trim_galore/ (free)

3. HISAT2 (version 2.1.0) [6]; http://daehwankimlab.github.io/hisat2/ (free)

4. featureCounts / Subread (version 2.0.3) [7]; http://subread.sourceforge.net (free)

5. samtools (version 1.23.1) [8]; http://www.htslib.org/ (free)

6. R (CRAN R project, version 4.4.3); https://www.r-project.org (free)

7. DESeq2 (version 1.38.3) [9]; https://bioconductor.org/packages/release/bioc/html/DESeq2.html (free)

8. Fiji (ImageJ; National Institutes of Health, USA; free)

9. Chicken reference genome and annotation: GRCg6a (Ensembl, GCA_000002315.5)

10. Dataset: PIC-RNA-seq of HH14 and HH19 chicken limb bud progenitor regions (deposited in GEO; accession numbers GSE342209)

11. The code used in this protocol has been deposited to GitHub: https://github.com/seungjunekwon/PIC-RNA-Seq_Chick_Limb (accessed July 21, 2026).

## Procedure

**A. Harvesting and dissecting the embryo**

1. Incubate fertilized chicken eggs on their side in an incubator at 38.5 °C for 2 and 3.5 days, to obtain Hamburger-Hamilton (HH) [10] 14 and 19 embryos, respectively. Confirm the developmental stage of each embryo according to the morphological criteria of Hamburger and Hamilton, rather than incubation time alone.

2. After the incubation, make a hole at the pointed end of the egg using curved scissors and remove approximately 3 mL of albumen using a 10 mL syringe fitted with an 18G × 1.5” needle.

3. Open a window (~2 cm in diameter) in the upper surface of the eggshell with curved scissors.

4. Harvest an embryo using a perforated spoon and transfer it into a handmade silicone-bottom dish filled with autoclaved 1× PBS at room temperature (RT).

5. Dissect the forelimb bud region by cutting the anterior and posterior edges using fine forceps (Figure 1A, B).

**Figure 1.**
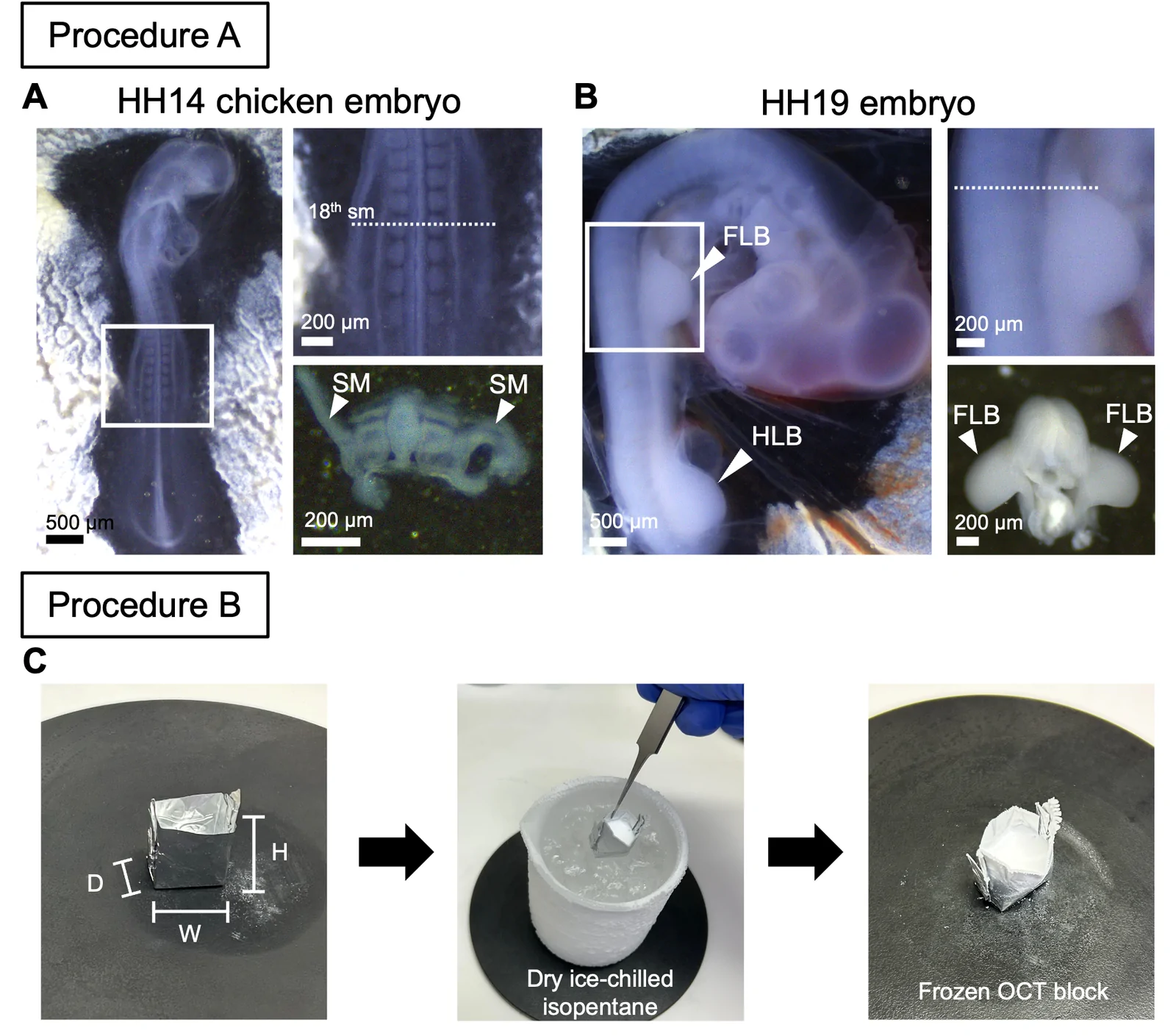
Chicken embryos at the limb bud stage and tissue embedding. ( (A) HH14 embryo. The boxed region indicates the prospective forelimb region to be dissected. The upper-right panel shows the dissection level at the 18^th^ somite, and the lower-right panel shows a transverse section at this level. SM, somatopleural mesoderm. (B) HH19 embryo. The boxed region indicates the forelimb bud region to be dissected. The upper-right panel shows the dissection level near the anterior end of the forelimb bud, and the lower-right panel shows a transverse section at this level. FLB, forelimb bud; HLB, hindlimb bud. (C) Embedding of the sample in OCT compound using an aluminum mold (approximately 10 × 15 × 15 mm; depth × width × height). Scale bars: 500 μm and 200 μm.

**B. Embedding and flash-freezing**

1. Prepare handmade aluminum foil cryomolds and half-fill them with OCT compound (Figure 1C).

2. Transfer the dissected forelimb bud region into the cryomold using a perforated spoon and gently mix twice to allow the tissue to be fully immersed in the OCT compound.

3. Prepare a 100 mL beaker containing crushed dry ice (pieces approximately 1–2 cm in size) and place it in a fume hood.

4. Pour isopentane into the beaker multiple times, as isopentane evaporates easily, until the beaker is approximately 80% filled with the dry ice–isopentane mixture.

5. Wait for at least 5 min until the isopentane bubbles subside. During this period, properly orient the tissue within the OCT compound using forceps (Figure 1A, B; anterior side touching the bottom surface of the mold perpendicularly).

6. Hold the cryomold with forceps and place it into the beaker to flash-freeze the sample. Ensure that the upper half of the mold does not enter the dry ice to prevent isopentane from entering the cryomold.

7. Wait approximately 30 s until the OCT compound is completely frozen and turns white; then, store the embedded tissue immediately at -80 °C (Figure 1C).

*Note: Embedded samples or cryosections stored at -80 °C for extended periods can still be processed; however, prolonged storage may reduce RNA integrity.*

**C. Cryosectioning**

*Note: See Figure 2 for sections C–O.*

1. Transfer embedded samples to the cryostat. To preserve RNA integrity, keep the cryomolds on ice during transfer if necessary.

2. Cryosection the embedded tissue at a thickness of 10 μm using a cryostat maintained at -10 to -15 °C.

3. Mount sections onto slides. Prepare approximately five serial sections for HH14 and three serial sections for HH19 samples to allow selection of an intact section containing an appropriate ROI. For each biological replicate, only one section from one embryo is used for PIC-RNA-seq. The remaining sections serve as backups.

*Note: Because some sections may be lost during subsequent procedures, particularly with the smaller HH14 samples, we recommend preparing one backup slide for each sample.*

4. Keep the slides on ice if necessary, then store them at -80 °C.

**Figure 2.**
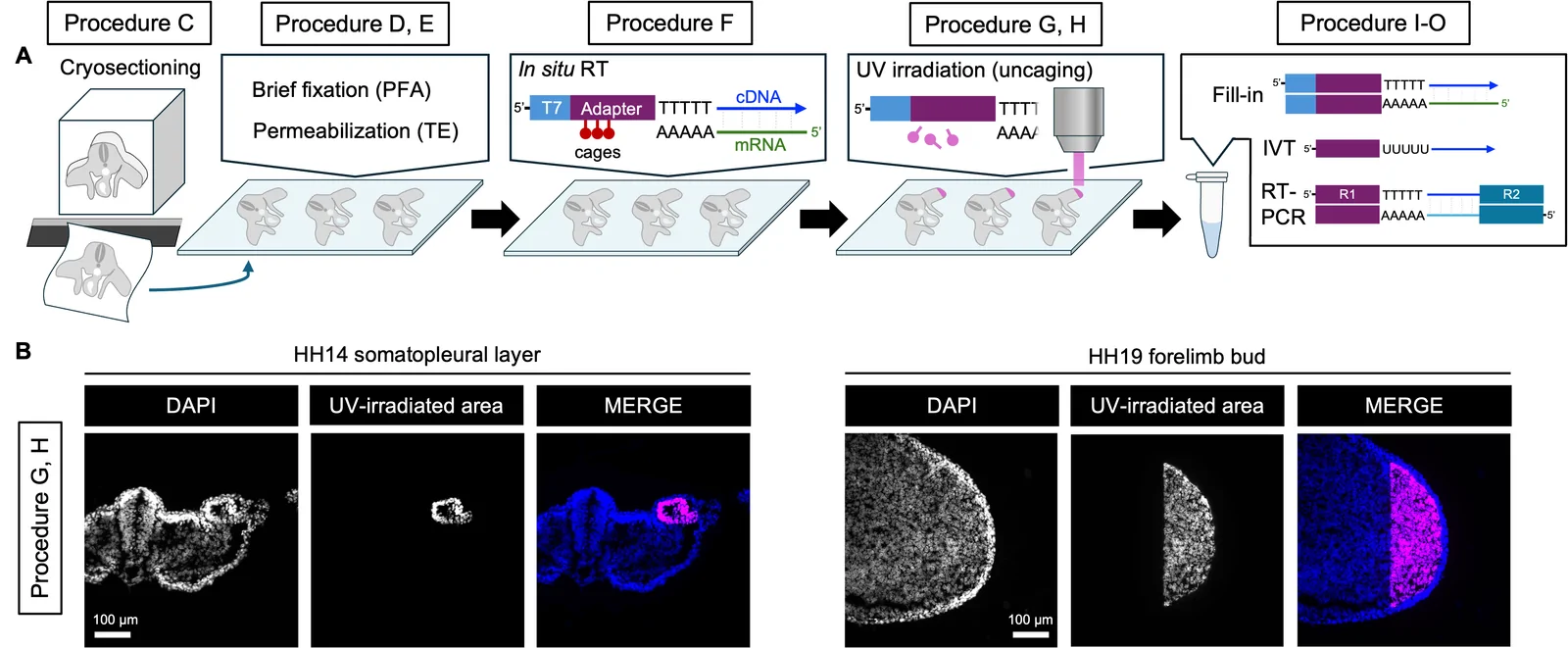
Workflow of the PIC-RNA-seq library preparation. (A) Workflow of the PIC-RNA-seq procedure from cryosectioning to RT-PCR. (B) Representative image showing the UV-irradiated area (magenta). Scale bar: 100 μm.

**D. Post-fixation of tissue sections**

*Note: Fresh-frozen sections are lightly post-fixed to retain tissue architecture before the enzymatic steps.*

1. Encircle each section with a hydrophobic barrier using a PAP pen. A 1 × 1 cm barrier holds ~50 μL of liquid without leakage. Scale the volume to the barrier size.

2. Drop 50 μL of PBS onto the section and aspirate. Repeat once.

3. Apply 50 μL of freshly prepared 4% PFA in PBS and leave at RT for 10 min.

4. Aspirate the fixative and rinse the section twice with 50 μL of PBS.

**Caution:** Handle PFA in a fume hood.

**Critical:** Keep fixation brief. Chicken embryonic tissue is fragile, and over-fixation cross-links mRNA excessively and lowers in situ RT efficiency (see General note 2).

**E. Permeabilization**

*Note: Heat-based TE permeabilization relaxes protein–RNA interactions and improves primer access, following the route optimized for fresh-frozen sections.*

1. Aspirate PBS from the sections and immerse the slides in TE buffer (pH 8.0) that has been prewarmed to 70 °C in a polypropylene staining jar. Incubate in a hybridization oven for 1 h.

2. Transfer the slides to a staining jar filled with chilled PBS and leave for at least 3 min until fully cooled.

3. Line the bottom of a humidified chamber with a paper towel and add 40 mL of PBS to equilibrate the vapor pressure.

**Critical:** Humidify with PBS, not water. Water vapor is drawn into the higher-salt droplets on the section and causes leakage past the hydrophobic barrier during long incubations.

4. Remove the slides from PBS, aspirate the excess, and place them in the humidified chamber.

5. Wash the sections twice with 50 μL of PBS.

**F. In situ reverse transcription with NPOM-caged RT primer**

*Notes:*

*1. mRNAs are reverse-transcribed in place using a photo-caged, barcoded RT primer. The NPOM cages block extension products from being amplified later until they are released by UV at the region of interest (ROI).*

*2. Each biological replicate is prepared on its own section and receives a primer with a distinct barcode. In this protocol, four replicate sections per group (HH14 and HH19) are carried through sections D–H in parallel and combined at cell lysis (section I).*

*3. The primer carries a T7 promoter, an Illumina adapter, a sample barcode, a UMI, and an oligo-dT tract, with NPOM-caged dT residues (sequence per [2]). Order it at OPC grade, not HPLC, because the UV used in HPLC size evaluation would prematurely uncage the NPOM groups. Store dried and light-shielded at −20 °C. A working stock at 500 ng/μL in nuclease-free water is also stored light-shielded.*

**Critical:** NPOM is stable under ordinary room lighting, but avoid prolonged light exposure of caged primers and of sections during long incubations. Primers carrying distinct barcodes are used so that the samples are pooled after cell lysis (step I4). Here, four barcodes (CATGAG, AGGATC, AGTGCA, TCGAAG) were used, one per biological replicate.

1. Prepare the NPOM-caged RT primer mix (see Recipe 3).

2. After a brief spin (1,400× *g*, 2 s, RT), heat the tube at 65 °C for 5 min in a thermal cycler to relax primer secondary structure, and immediately chill on ice for at least 2 min.

3. Prepare the first-strand mix (see Recipe 4).

4. Combine 6 μL of primer mix (step F2) with 4 μL of first-strand mix (step F3) in a 0.2 mL 8-strip tube by gentle pipetting.

5. Pipette the 10 μL reaction directly onto the section and confirm that the entire tissue is covered by the liquid.

**Critical:** Keep the humidified chamber balanced with PBS so that the reaction does not dry out during the 1-h incubation.

6. Incubate the slides in the PBS-humidified chamber at 42 °C for 1 h in the hybridization oven.

7. Immerse the slides in PBS prewarmed to 70 °C for 10 min to stop the RT reaction, and cool them in chilled PBS for at least 3 min.

**Pause point:** Sections can be held at 4 °C for up to 3 days under light-shielded conditions.

8. Aspirate the excess PBS, return the slides to the humidified chamber, and wash twice with 50 μL of PBS.

**G. Nuclear staining for ROI visualization**

*Note: ROIs are delineated from the tissue architecture outlined by nuclear staining with Nuclear Violet LCS1 (hereafter, the nuclear stain). This dye has two properties relevant to PIC: it can be excited at 426–450 nm (i.e., above the ~425 nm threshold below which the NPOM would be uncaged), so nuclei can be observed without triggering uncaging, and it is additionally excited by 365 nm UV, so the same signal also reports which cells have been irradiated.*

1. Aspirate PBS and apply 50 μL of the nuclear stain diluted 1:500 in PBS. Incubate at RT for 30 min in the humidified chamber.

2. Aspirate the staining solution and wash three times with 50 μL of PBS.

**H. UV irradiation and uncaging by DMD**

*Note: The caged primers within the ROI are released by patterned 365 nm light delivered through the DMD.*

1. Aspirate PBS and mount a 15 mm round coverslip with 10 μL of SlowFade Diamond, avoiding air bubbles.

2. Under the fluorescence microscope, acquire an image of the nuclear stain using an excitation wavelength longer than 425 nm (to avoid unintended uncaging) and identify the ROI:

a. HH14: Select the somatopleural layer of the lateral plate mesoderm (prospective forelimb field, around the level of the 18th somite) as the ROI. The irradiated ROIs had an average area of approximately 6,700 μm^2^ and contained ~80 nuclei (n = 4 biological replicates). The ROI extended approximately 180 μm laterally from the edge of the nephric duct.

b. HH19: Select the forelimb bud mesenchyme as the ROI, excluding the surface ectoderm. The irradiated ROIs had an average area of approximately 22,400 μm^2^ and contained ~280 nuclei (n = 4 biological replicates). The mesenchymal ROI extended approximately 90 μm proximally from beneath the apical ectodermal ridge.

c. ROI selection and UV irradiation are performed using a 20× objective lens.

d. ROI area and the number of nuclei within each ROI are quantified using Fiji (ImageJ).

3. Overlay the DMD control window on the live image and outline the ROI.

4. Switch on the 365 nm LED and irradiate the ROI for 3 min.

5. Capture an image of the irradiated area (the nuclear stain is also excited by 365 nm and marks the illuminated region) to document the ROI.

**Critical:** Our setup was a Leica DM6B microscope fitted with a Mightex Polygon1000-G DMD and a Prizmatix UHP-F-365 LED. Other microscope/DMD/LED combinations require re-optimization of irradiation time, which can be checked by qPCR without sequencing. Specifically, tissues are irradiated across a range of UV intensities and exposure times, and the extent of uncaging is assessed by TaqMan qPCR with primers against the T7 promoter and the *GAPDH* 3′ untranslated region. The lowest dose at which the qPCR signal plateaus is taken as the optimal uncaging condition.

*Note: When applying this protocol to a new tissue, process a non-irradiated section in parallel and confirm that almost no library is obtained. Alternatively, irradiate an anatomically distinct adjacent region on a serial section and confirm that the expected region-restricted markers differ between the two.*

**I. Cell lysis and collection**

*Note: Whole-cell lysates are recovered after uncaging, and the four barcoded sections are pooled into a single tube.*

1. Float off the coverslip with 300 μL of PBS and remove it with tweezers. Wash the section twice with 50 μL of PBS.

2. Apply 40 μL of the ProK lysis solution (see Recipe 12) onto the section and collect the lysate and debris into a 1.5 mL tube while scraping the tissue off with a pipette tip.

**Critical:** Recover the whole lysate thoroughly. Material left on the slide lowers the yield.

3. Apply a second 40 μL volume of the ProK lysis solution to the same section, and again scrape and collect into the same tube (80 μL per section).

4. Pool the barcoded sections. Carry out steps I1–3 for all four sections of the group, each reverse-transcribed with a distinct barcode in section F, and collect them into a single tube. The pooled lysate is ~320 μL (80 μL per section × 4 sections).

5. Incubate the pooled lysate at 55 °C for 1 h on a heat shaker.

**Pause point:** The protocol can be paused here. Samples can be stored at −30 °C for at least 1 week, light-shielded.

**J. Purification of cDNA:mRNA hybrids**

*Note: The following purification is performed on the pooled lysate (~320 μL = 80 μL per section × 4 sections).*

1. Add 5 volumes of buffer PB (MinElute PCR Purification kit, ~1,600 μL) to the pooled lysate (~320 μL) and mix. The total volume is ~1,920 μL.

2. Because a MinElute column holds ≤750 μL per load, purify the entire sample on a single column by loading it in successive aliquots (about three loads in total): apply ≤750 μL to the column, centrifuge at 15,300× *g* for 1 min at RT, and discard the flowthrough. Repeat with the remaining sample until all ~1,920 μL has passed through the same column.

3. Wash with 750 μL of buffer PE (15,300× *g* for 1 min at RT). Discard the flowthrough and spin once more (2 min) to dry the membrane.

4. Transfer the column to a fresh 1.5 mL tube, add 17 μL of nuclease-free water, let stand 1 min, and elute by centrifugation (15,300× *g*, 2 min, RT).

**Pause point:** The protocol can be paused here. Samples can be stored at -20 °C for up to 1 month, light-shielded.

**K. Second-strand DNA synthesis**

*Note: Nick-translation second-strand synthesis fills in the T7 promoter only on cDNAs derived from the irradiated ROI, making them competent for linear amplification.*

1. Prepare the second-strand mix (see Recipe 5).

2. Combine 5 μL of the mix with 15 μL of purified hybrids (step J4) in a 0.2 mL 8-strip tube.

3. After a brief spin, incubate at 16 °C for 2 h in a thermal cycler and immediately place the tube on ice.

4. Mix AMPure XP beads and beads binding buffer (see Recipe 6) at 1:5 and vortex.

5. Add 24 μL of the bead mix (1.2× the sample volume) to the reaction and vortex.

6. After a brief spin, let the tube stand at RT for 15 min and place it on a magnetic stand for ≥5 min until the beads are fully collected.

7. Remove the supernatant without disturbing the beads.

8. Wash twice with 200 μL of 80% ethanol (30 s each), removing the supernatant each time. After the second wash, spin the tube down, return it to the magnet for 1 min, remove residual ethanol, and air-dry for 2 min.

**Critical:** Ensure no ethanol remains.

9. Resuspend the beads in 6.4 μL of nuclease-free water and let them stand for ≥2 min off the magnet.

**L. In vitro transcription (IVT)**

*Note: cDNAs from the ROI are linearly amplified into antisense RNA (aRNA).*

1. Prepare the IVT mix (see Recipe 7).

2. Add 9.6 μL of IVT mix directly to the bead suspension (step K9) in a 0.2 mL 8-strip tube.

3. After a brief spin, incubate at 37 °C for 17 h and immediately place the tube on ice.

**M. aRNA purification**

1. Add 1 μL of TURBO DNase, spin down, incubate at 37 °C for 20 min, and immediately place the tube on ice.

2. To stop the DNase reaction, add 8.5 μL of nuclease-free water and 2.75 μL of 0.5 M EDTA (pH 8.0) and vortex.

3. Spin down, place on the magnetic stand for 5 min, and collect 28 μL of supernatant into a fresh tube.

4. Add 50.4 μL of RNAClean XP beads (1.8× the volume) and vortex. After standing at RT for 10 min, place the tube on the magnet for ≥5 min.

5. Remove the supernatant and wash twice with 200 μL of 70% ethanol (30 s each). After the second wash, spin the tube down, return it to the magnet, remove residual ethanol, and air-dry for 2 min.

6. Resuspend in 6 μL of nuclease-free water, let it stand for ≥2 min, spin down, and place on the magnet for 5 min.

7. Collect 4.5 μL of supernatant into a fresh 0.2 mL 8-strip tube.

**Pause point:** The protocol can be paused here. Samples can be stored at −80 °C for up to 1 month.

**N. Reverse transcription of aRNA**

*Note: aRNA is reverse-transcribed with a random primer carrying the Illumina Read2 sequence.*

1. Prepare the aRNA–N6 primer mix (see Recipe 8).

2. After a brief spin, heat at 65 °C for 5 min to relax secondary structure and immediately chill on ice.

3. Prepare the reverse transcription mix (see Recipe 9).

4. Add 4 μL of RT mix to the primer mix, pipette gently, spin down, and incubate at 25 °C for 10 min followed by 42 °C for 1 h.

**Pause point:** The protocol can be paused here. Samples can be stored at 4 °C for up to 1 day.

**O. Library amplification by PCR**

1. Prepare the library PCR mix (see Recipe 11).

2. Spin down and amplify using the cycling conditions shown in Table 1.

**Table 1.** PCR cycling conditions for library amplification

Step	Temperature	Time	Cycles
Initial denaturation	98 °C	30 s	1
Denaturation	98 °C	10 s	11
Annealing	60 °C	30 s	(11 cycles)
Extension	72 °C	30 s	(11 cycles)
Final extension	72 °C	10 min	1
Hold	4 °C	∞	n/a

**Critical:** Increase to ~15 cycles when few cells were irradiated and low yield is expected. Excessive cycles over-amplify and saturate UMIs.

**Pause point:** The protocol can be paused here. Samples can be stored at −20 °C for up to 1 month.

3. Add 1 μL of RNase A (10 mg/mL), spin down, and incubate at 37 °C for 30 min.

**P. Size selection of the sequencing library**

*Note: Two rounds of double-sided bead selection recover the 200–400 bp library.*

**Critical:** Perform at least two rounds. Repeat further if the size falls outside 200–400 bp.

**Round 1**

1. Bring the PCR product to 50 μL with nuclease-free water.

2. Add 32.5 μL of AMPure XP beads (0.65×), vortex, stand 15 min, and place on the magnet for ≥5 min.

3. Transfer the supernatant to a fresh tube (large fragments are discarded on the beads).

4. Add 12.5 μL of AMPure XP beads to the supernatant, vortex, let it stand for 10 min, and place on the magnet for ≥5 min.

5. Remove the supernatant. Wash twice with 200 μL of 80% ethanol (30 s each). Dry as in step K8.

6. Resuspend in 32 μL of nuclease-free water, let it stand for ≥2 min, place on the magnet for 5 min, and collect 30 μL.

**Round 2**

7. Repeat the double-sided selection on the 30 μL sample using AMPure XP beads at 19.5 μL, followed by 7.5 μL, washing and drying as above.

8. Resuspend in 15 μL of nuclease-free water and collect 12 μL of the final library.

**Pause point:** The protocol can be paused here. Samples can be stored at -20 °C for up to 6 months.

**Q. Library quantification**

1. Load 2 μL of the library on a High Sensitivity D5000 Screen Tape and run on the TapeStation per the manufacturer's instructions.

**Critical:** The expected peak is 200–400 bp. If the size is off-target, repeat size selection (section P). See Troubleshooting.

**R. Sequencing**

1. Perform paired-end sequencing on an Illumina NovaSeq 6000. Read 1 reads the UMI and barcode, and Read 2 reads the cDNA.

a. Read 1: 19 bp (UMI, barcode, and additional bases).

b. Read 2: 81 bp (cDNA).

c. PhiX spike-in: ~5%.

d. Target depth: approximately 2 million reads per sample.

2. Confirm run quality and per-sample read counts before proceeding to Data analysis.

## Data analysis

Sequencing reads are processed as described below with a UMI-based workflow, following the PIC data-analysis procedure [1,2] (Figure 3). Basic Linux/command-line and R proficiency is assumed.

**Figure 3.**
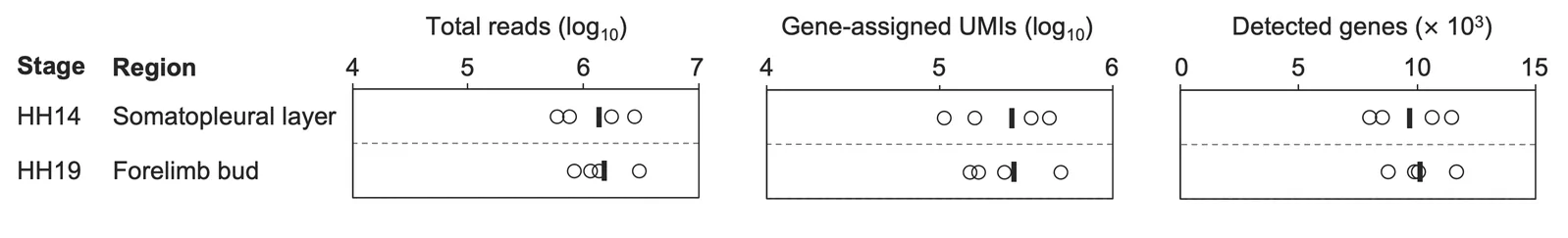
Sequencing metrics of PIC-RNA-seq. Numbers of total reads, gene-assigned reads, unique molecular identifiers (UMIs), and detected genes are shown for each PIC-RNA-seq sample. Black vertical lines indicate the mean of each metric across replicates.

1. Barcode/UMI extraction: Extract cell barcodes and UMIs from Read 1 with UMI-tools:

umi_tools extract -I read1.fastq --read2-in=read2.fastq \

--bc-pattern=NNNNNNCCCCCC --read2-stdout –log2stderr > sample.bc_aligned.fastq

2. Adapter trimming: Remove Illumina universal adapters with Trim Galore!:

trim_galore -a GATCGTCGGACT –basename sample sample.bc_aligned.fastq

3. Alignment: Map reads to the chicken reference genome (GRCg6a; Ensembl, GCA_000002315.5) with HISAT2.

hisat2 -x GRCg6a_index -U sample_trimmed.fq -S sample.sam

samtools sort -o sample.bam sample.sam

samtools index sample.bam

4. Gene assignment and counting: Assign reads to genes with featureCounts and deduplicate by UMI:

featureCounts -a GRCg6a.gtf -R BAM sample.bam

samtools index sample.featureCounts.bam

umi_tools count --method=unique --per-gene --per-cell \

--gene-tag=XT -I sample.featureCounts.bam -S sample.counts.tsv

5. Normalization, PCA, and DEG: Import the gene × sample UMI count matrix into DESeq2. Apply the variance-stabilizing (vsd) transform and run principal component analysis with plotPCA (Figure 4A). Identify differentially expressed genes (DEGs) between HH14 and HH19 with DESeq2 (Wald test, adjusted p-value by Benjamini–Hochberg) (Tables S1 and S2). The contrast was HH19 versus HH14 (HH14 as the reference level); therefore, a positive log_2 _fold change indicates higher expression in HH19. DEGs were defined by an adjusted p-value < 0.05 with no fold change cutoff.

library(DESeq2)

# umi_counts is the gene (rows) x sample (columns) UMI count matrix,

# whose column names match the row names below.

def <- data.frame(

row.names = c("hh14_1", "hh14_2", "hh14_3", "hh14_4",

"hh19_1", "hh19_2", "hh19_3", "hh19_4"),

group = c("hh14", "hh14", "hh14", "hh14",

"hh19", "hh19", "hh19", "hh19")

)

def$group <- relevel(factor(def$group), ref = "hh14")

dds <- DESeqDataSetFromMatrix(countData = umi_counts, colData = def, design = ~ group)

dds <- DESeq(dds)

res <- results(dds, contrast = c("group", "hh19", "hh14"))

vsd <- vst(dds); plotPCA(vsd, intgroup = "group")

normcount <- counts(dds, normalized = TRUE)

6. Generate a volcano plot with R libraries ggplot2 and ggrepel (selected genes were labeled; Figure 4B).

7. Generate box plots of DEGs using DESeq2 size-factor-normalized counts (Table S2) with R libraries ggplot2, tidyr, and dplyr (Figure 4C).

Four biological replicates per group were analyzed (HH14 and HH19 forelimb bud, n = 4 each). The per-sample QC metrics (total reads, gene-assigned UMIs, detected genes, UMIs/genes, assigned/UMI) are summarized in Figure 3, and the PCA and DEG results are shown in Figure 4.

**Figure 4.**
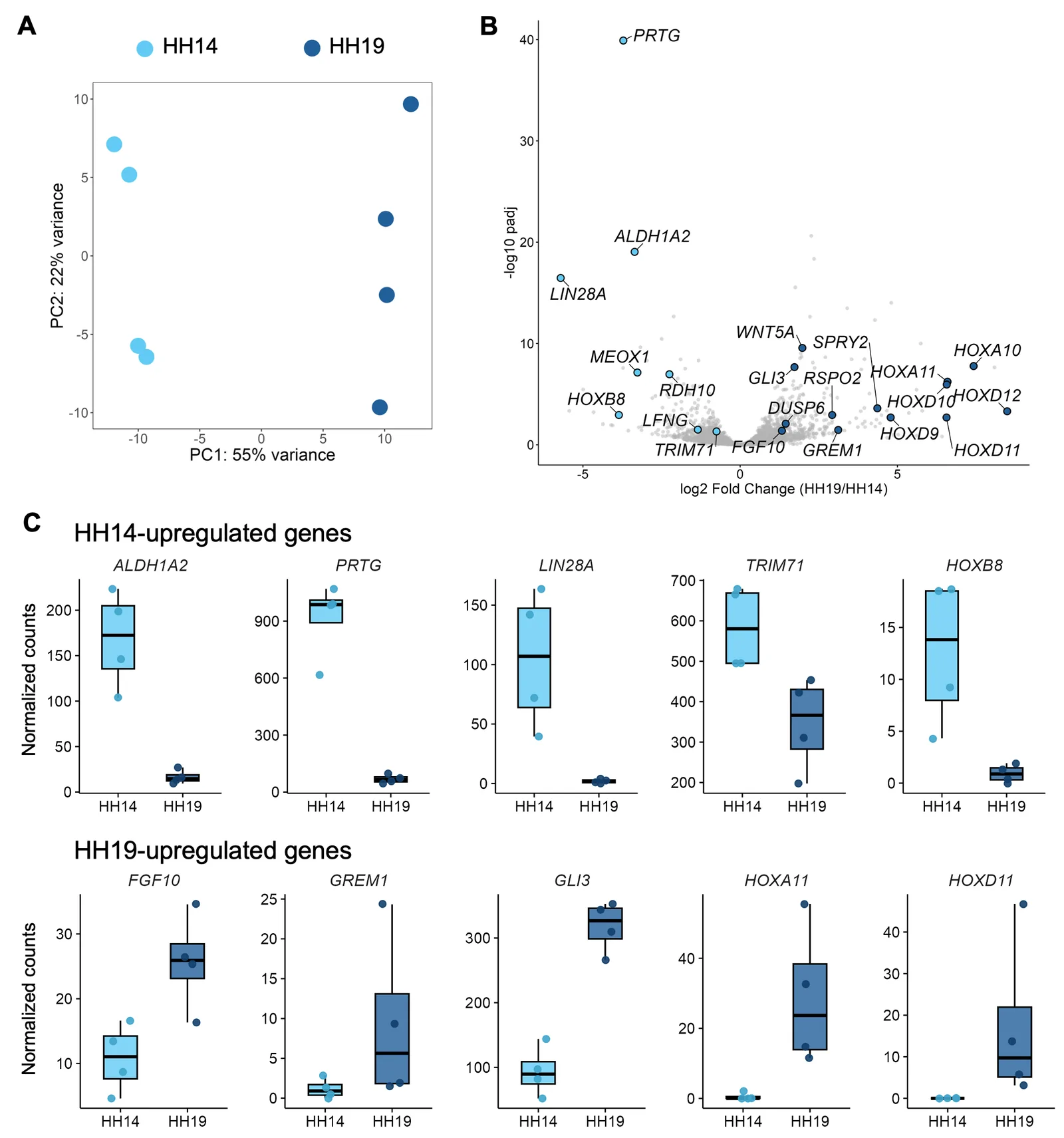
Downstream transcriptomic analyses of HH14 and HH19 forelimb buds. (A) PCA analysis showing clear separation between Hamburger–Hamilton (HH) 14 and 19 chicken samples along the PC1 axis. (B) Volcano plot comparing HH14 sLPM and HH19 forelimb bud samples. Genes with values under the threshold are colored gray. Among 508 differentially expressed genes (DEGs), representative ones are labeled. Differential expression analysis was performed using DESeq2 (p-value obtained from the Wald test), and adjusted p-values (using the Benjamini–Hochberg method) are shown in the volcano plots. (C) Box plots showing the differential expression of HH14- and HH19-upregulated genes. Box plots indicate the median (center line), 25th and 75th percentiles (box), and whiskers extending to the minimum and maximum values within 1.5× the interquartile range. Individual data points are overlaid (n = 4 each; biological replicates from different embryos).

## Validation of protocol

This protocol applies the previously reported PIC-RNA-seq approach to chicken sLPM and LPCs [2,4]. The representative results below, provided directly in this section, show that the protocol yields region- and stage-specific transcriptomes whose differentially expressed genes agree with known limb-development biology.

1. Library and sequencing QC (Figure 3): All samples yielded the expected number of sequencing reads. In practice, the key indicators of a successful library are the number of detected genes (coverage) and the number of UMIs per detected gene, the latter of which determines the dynamic range available for differential expression. Across HH14 (n = 4) and HH19 forelimb bud (n = 4), ~8,000–12,000 genes were detected per replicate, with ~10–50 UMIs per gene (~1 × 10 to 5 × 10 gene-assigned UMIs).

2. Global separation by stage (Figure 4A): vsd-transformed data separated HH14 and HH19 samples along PC1 (55% of variance), indicating that the photo-isolated transcriptomes capture stage-specific programs.

3. Stage-specific markers as DEGs (Figure 4B, C): DEG analysis (DESeq2, adjusted p < 0.05) identified 192 genes higher in HH14 (log_2 _fold change < 0) and 316 higher in HH19 (log_2 _fold change > 0). HH14 (early limb field)-upregulated genes included *ALDH1A2, PRTG, LIN28A, TRIM71*, and *HOXB8*, whereas HH19 (limb bud)-upregulated genes included *FGF10, GREM1, GLI3, HOXA11*, and *HOXD11*, consistent with the transition from a proximal/undifferentiated field to a patterned, outgrowing limb bud.

4. ROI specificity (Figure 4C): *ALDH1A2* is expressed throughout the lateral plate mesoderm at HH14 and is strongly reduced in the limb bud as it forms, while remaining high in the flank immediately adjacent to the bud [10]. Accordingly, *ALDH1A2* was 10.3-fold lower in the HH19 forelimb bud than in the HH14 sLPM (adjusted p = 9.0 × 10^–20^), as expected if the irradiated area was confined to the bud and did not extend into the flank.

## General notes and troubleshooting

**General notes**

1. Keep fixation brief. A short PFA fixation preserves tissue morphology while avoiding the excess mRNA cross-linking that would otherwise lower in situ RT efficiency. Do not extend the fixation time.

2. Cell number: A sufficient transcriptome is typically obtained from ROIs containing approximately 80 or more nuclei. Small ROIs give lower yields. Consider enlarging the ROI or increasing the number of irradiated cells by pooling multiple barcoded sections (sections F and I).

3. Multiple ROIs: Several ROIs on one section can be irradiated together, but they are recovered as a single pooled sample and cannot be separated afterward, so irradiate one ROI type per barcode.

4. Light management: Keep caged primers and sections shielded from light during extended incubations (in situ RT, nuclear staining, lysis, second-strand synthesis) to prevent stray uncaging.

5. Replicates: Use at least 3–4 biological replicates per group for DEG detection. More replicates increase statistical power for small or similar ROIs.

6. Batch design: In this protocol, samples from each stage were pooled into a separate library, so the developmental stage is potentially confounded with the library-preparation batch at the library level (replicate-level resolution is retained through barcoding). Where batch effects are a concern, distribute the replicates of each group across multiple libraries rather than pooling each stage into a single library.

**Troubleshooting**

**Table:** 

Problem	Possible cause	Solution
Cryosections contain holes	Isopentane entered the cryomold, causing the sample to freeze unevenly	Before freezing samples in a cryomold in section B, wait more than 10 min until the isopentane bubbles have completely subsided
No or low library yield	Degraded RNA	Store frozen sections at −80 °C and process them promptly; minimize handling time
	Over-fixation cross-linking mRNA	Keep the PFA fixation as brief as specified in section D
	Too few cells in the ROI	Irradiate ≥100 cells or pool multiple barcoded sections (sections F, I)
	Insufficient PCR amplification	Increase the number of PCR cycles (section O)
	Insufficient uncaging	Optimize LED power/irradiation time. The optimal UV irradiation condition can be estimated by qPCR without sequencing (section H)
Unexpected background/high nonspecific signal	Unintended uncaging	Order OPC-grade (not HPLC) caged primer and keep samples light-shielded through all long incubations
Library size outside 200–400 bp	Incomplete size selection	Repeat size selection (section P) and add further rounds as needed
Few DEGs detected	Low replicate number/shallow depth	Increase replicates and/or sequencing depth and check per-sample QC (Figure 3)
Weak or absent nuclear staining	Insufficient staining or wrong excitation	Confirm nuclear-stain dilution and incubation and image only at >425 nm excitation to avoid uncaging

## Supplementary information

The following supporting information can be downloaded here:

1. Table S1. List of DEGs between chicken limb bud progenitors of HH14 and HH19

2. Table S2. DESeq2 size-factor-normalized count table of HH14 and HH19 chicken limb bud progenitors
